# Bootstrapping Virtual Bipedal Walkers with Robotics Scaffolded Learning

**DOI:** 10.3389/frobt.2021.702599

**Published:** 2021-09-08

**Authors:** Jiahui Zhu, Chunyan Rong, Fumiya Iida, Andre Rosendo

**Affiliations:** ^1^ Living Machines Laboratory, School of Information Science and Technology, ShanghaiTech University, Shanghai, China; ^2^ Bio-Inspired Robotics Laboratory, Department of Engineering, University of Cambridge, Cambridge, United Kingdom

**Keywords:** robotics scaffolded learning, bootstrapping, bio-inspired learning, bio-inspired robotics, bipedal locomotion

## Abstract

We reach walking optimality from a very early age by using natural supports, which can be the hands of our parents, chairs, and training wheels, and bootstrap a new knowledge from the recently acquired one. The idea behind bootstrapping is to use the previously acquired knowledge from simpler tasks to accelerate the learning of more complicated ones. In this paper, we propose a scaffolded learning method from an evolutionary perspective, where a biped creature achieves stable and independent bipedal walking while exploiting the natural scaffold of its changing morphology to create a third limb. The novelty of this work is speeding up the learning process with an artificially recreated scaffolded learning. We compare three conditions of scaffolded learning (free, time-constrained, and performance-based scaffolded learning) to reach bipedalism, and we prove that a performance-based scaffold, which is designed by the walking velocity obtained, is the most conducive to bootstrap the learning of bipedal walking. The scope of this work is not to study bipedal locomotion but to investigate the contribution from scaffolded learning to a faster learning process. Beyond a pedagogical experiment, this work presents a powerful tool to accelerate the learning of complex tasks in the Robotics field.

## 1 Introduction

Scaffolding is a learner-centered teaching method based on the constructivist learning theory, aiming at cultivating the problem-solving ability and autonomous learning ability of the students. Pedagogy explains it as providing small-step clues or hints (scaffolds) for students to learn step by step to discover and solve problems gradually. This method leads to students mastering the knowledge to be learned, improving their problem-solving ability, and eventually growing into independent learners. Vygotsky, a famous psychologist in the former Soviet Union, derived this teaching idea from the “zone of proximal development” theory (Chaiklin, 2003). Pedagogical applications have actively used scaffolding to bootstrap knowledge (Quarles et al., 2009; Al Mamun et al., 2020), and some researchers tried to understand its associations with human locomotion: the ontogenetic development (Lungarella et al., 2003) of bipedal walking in human infants (Susa, 1981), and the mechanism of acquiring general motor skills and of human walking (Okamoto, 1985; Thelen et al., 1991). Besides, reaching bipedal locomotion (Wahde and Pettersson, 2002; Vukobratovic et al., 2012; Giardina and Mahadevan, 2021) during early childhood requires individuals to be strong enough to support their weight, stable enough to resist an ever oscillating center of gravity, and to move in a state of dynamic balance when the body alternates between the double support and the single support (Bril and Brenière, 1993; Owaki et al., 2013; Swan et al., 2020). Along these lines, the work from Hase and Yamazaki (1998) simulated a supported infant walking by applying linear springs and dampers to a bipedal walking model, which was controlled by a rhythm-generation mechanism called central pattern generator (Grillner and Wallen, 1985; Reil and Husbands, 2002). As this simulated infant learned to walk, it naturally became upright and prescinded from the spring support, but A. the mechanisms deciding the amount of/need for supports to be given or B. the benefits compared to the absence of such supports were not investigated.

**FIGURE 1:**
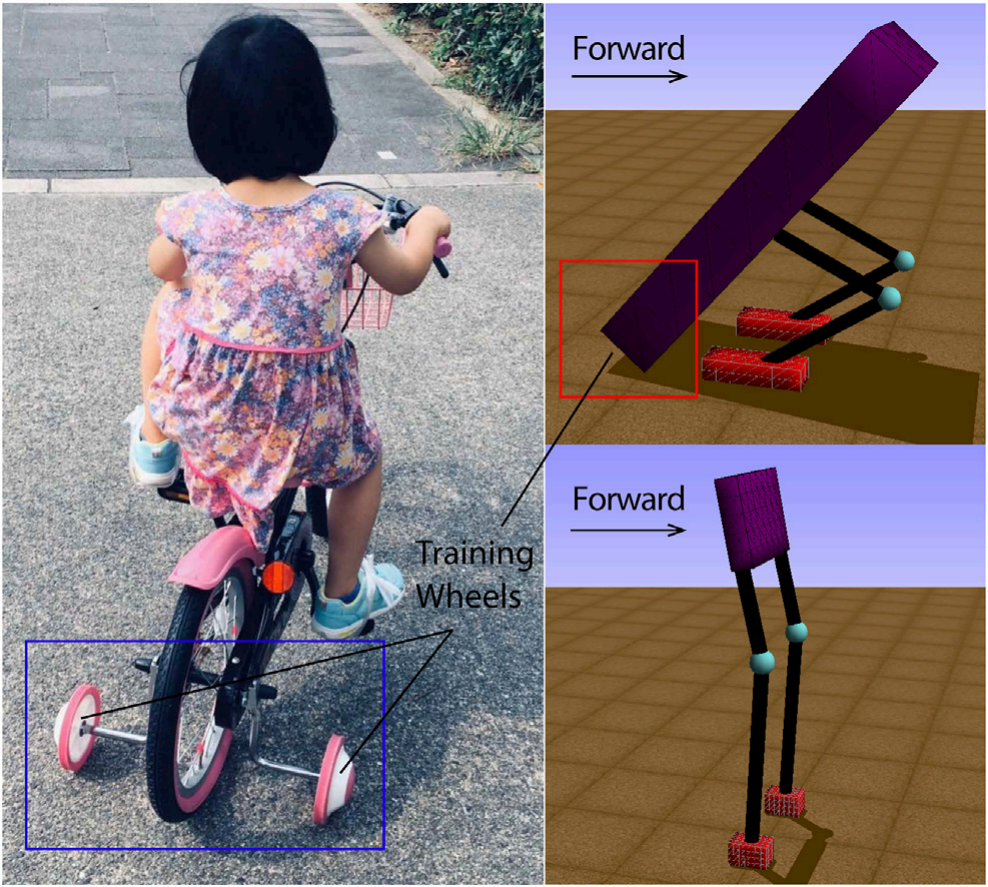
The general idea of scaffolded learning can be exemplified with a child on training wheels **(the left figure)**. The long body length of a biped creature can be adjusted to act as a tripod with shorter legs **(the upper right figure)**, sacrificing speed to gain stability, while a longer leg and short body would be optimal bipedalism **(the lower right figure)**.

In this article, we present a scaffolded learning method for bipedalism to bootstrap its ontogenetic development with gradual morphological and control changes (Owaki et al., 2008; Saar et al., 2017). Although AI and statistics usually have a specific meaning for bootstrap, the one that we used is more related to cognition, as seen in the works of Owaki and Ishiguro (2006); Kaipa et al. (2010); Schmidt et al. (2013); Giardina and Iida (2016); Howard et al. (2019). Bootstrapping is the concept of capitalizing the previously acquired skills to accelerate the learning of new skills on top of existing ones. Inspired by the mechanisms of a child learning to ride a bike with training wheels, as shown in Figure 1, our simulations consist of a two-legged creature with a long body that can be dragged through the floor as a tripod, providing a scaffold during the learning of bipedalism. Since our work is based on scaffolded learning, in the context of evolutionary robotics, we decided to choose genetic algorithms (Back, 1996; Chernova and Veloso, 2004) to evolve both morphology (the dimensions of the body, the leg and the foot) and control (virtual model control) of the creature. In the work of Karl Sims (Sims, 1994), genetic algorithms are used to generate the morphology of virtual creatures and neural systems for controlling their muscle forces, while they describe a system for creating virtual creatures. The difference is that our work is not creating virtual creatures for computer animation, but proposing a scaffolded learning method to bootstrap the learning of the bipedal walking. In addition, we define the fitness function as the walking distance traveled in 15 seconds divided by the leg length to maximize the forward walking speed of bipedal walkers. In our initial simulations, the creature tries to maximize its walking velocity while evolving freely, and later we introduce two evolving conditions where we force the creature to reduce its body length (abdicate from its scaffold) over fixed time intervals or once the creature makes gradual performance improvements. We show that the performance-based scaffold is superior to the time-constrained case and the free scaffolded case, as it allows the controller to be robust before becoming independent from the morphological support. We explain the effectiveness of the bootstrapping mechanism, draw parallels to robotic implementations of ontogeny (Vujovic et al., 2017), and propose a framework where real-world robots can use a similar approach to bootstrap knowledge (Rosendo et al., 2017). In *Materials and Methods*, we describe our adopted methods and present the performance metrics for our simulation, along with three scaffolded learning cases. We show the results in *Results* and discuss the implication of these results in *Discussion*. In *Conclusion*, we conclude our work.

## 2 Materials and Methods

### 2.1 Genetic Algorithm

Genetic Algorithms (GA) (Chernova and Veloso, 2004) uses simulated evolution to search for solutions to complicated problems. The algorithm adapts to select, recombine, and mutate processes on encoded genotypes, where they are leg lengths, body sizes, foot sizes, spring stiffness, damper viscosity, step offset, and stand offset, and evaluate the fitness of each individual to evolve it over generations. We adopt a subset of the generation containing the fittest individuals to create the new generation under an exploration-exploitation trade-off so that GA can find a globally optimal solution.

As the pseudo-code shown in Algorithm 1, we choose the population size *n* as 16, and the number of generations *G* as 4,000. At first, we create the initial population by the default parameter set and calculate their fitness to obtain the set of the fittest individuals *F*, while *F* has the same size as the population size. Then we create the new population by crossover and mutation with Gaussian distributions, while the individuals are from the fittest individual set. Here, we add Gaussian noise in order to ensure sensible results. Next, we evaluate the new individual one by one and compare them with the individual in *F*. If the fitness of the new individual is higher than the lowest one in *F*, the lowest individual in *F* will be replaced. If not, the next individual will conduct the same replacement until all the new individuals have been compared. After that, the algorithm will do the next loop of new generations. 
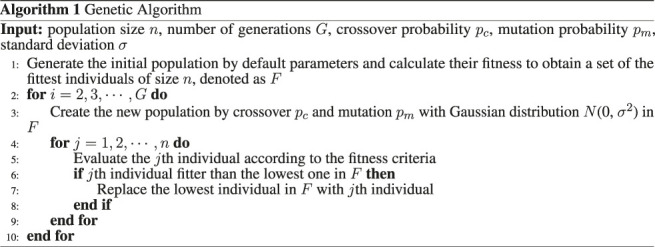


### 2.2 Virtual Model Control

Virtual Model Control (VMC), developed by Pratt et al. (1997), is a motion control framework that uses a desired virtual force, combined with the kinematics model (see 1) and its Jacobian (see 2), to generate desired joint torques on the stance leg (see 3). The combination of these joint torques creates the same output that the virtual force would have created, thereby creating the intended motion on the creature. Such forces can be emulated as products from many components, such as springs, dampers, masses, dissipative fields, or any other imaginable component.

**FIGURE 2:**
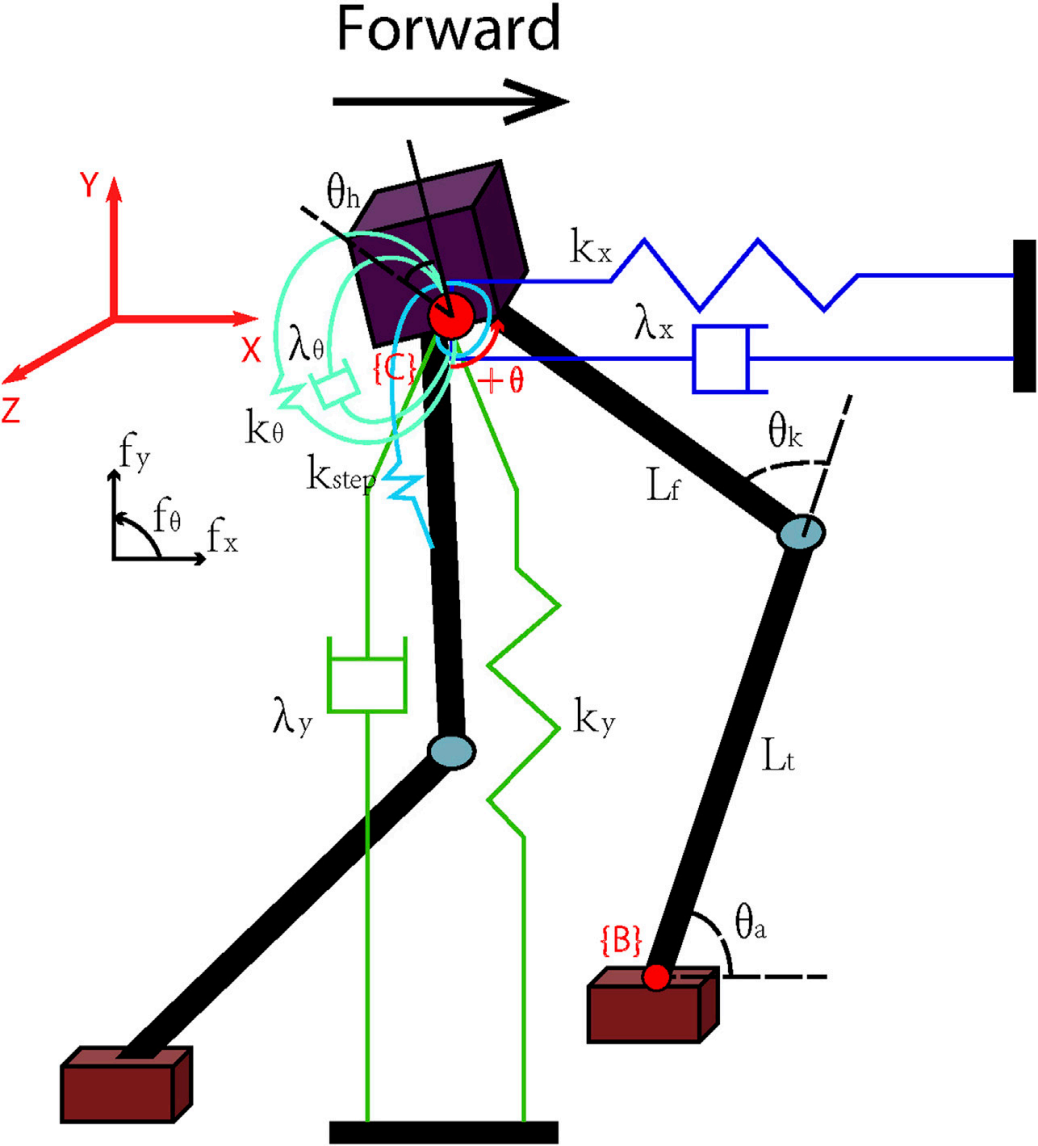
The virtual model in our simulation. We attach linear springs and dampers to the hip position of the individual as the granny walker mechanism, to maintain a constant height, and the dog-track bunny mechanism applies a virtual force in the forward horizontal direction to obtain the desired velocity. In addition, it has a torsional spring and a rotary damper acting on the hip joint to keep the upper body straight in the standing phase, while in the step phase, the hip joint only has a torsional spring with *k*_*step*_ spring stiffness to swing the leg. The wall which the spring and the damper are attached is virtual, and only exists to create the virtual force *f*_*x*_, *f*_*y*_, and *f*_*θ*_. Each component of the individual is a changeable genotype, such as the stiffness of the spring *k*_*x*_, *k*_*y*_, *k*_*θ*_, and *k*_*step*_, the viscosity of the damper *λ*_*x*_, *λ*_*y*_, and *λ*_*θ*_, the length of the femur *L*_*f*_ and the tibia *L*_*t*_, and three dimensional sizes of the torso and the foot.

Figure 2 shows the virtual model control for a single leg of the creature to perform a forward walking with horizontal, vertical, torsional springs, and dampers components. The horizontal spring and damper are used to create a virtual force in the horizontal direction, so the biped creature can move horizontally by the force. The wall which the spring and the damper are attached is virtual, and only exists to create the virtual force. The biped model can be shown in two dimensions or three dimensions. In order to let the reader more clearly see the posture change of bipeds during the walking learning, we decide to show the model in three dimensions. Table 1 lists the virtual model parameter used in our work. In addition, the mass is calculated depending on the body size, and the foot size, for instance, if the body length becomes longer, the body mass will proportionally increase. As for the moment of inertia, it is depending on the mass, the body size, and the foot size, so if the body length increases, the moment of inertia of the body will become larger. We defined the offset parameters *standOffset* in the standing phase and *stepOffset* in the step phase, which can control the natural length of the virtual springs, determined by the genetic algorithm, as shown in Table 1.

**TABLE 1:** Virtual Model Parameters.

Parameter	Description
*halfBodyX*	Half size of the body in *x* direction
*halfBodyY*	Half size of the body in *y* direction
*halfBodyZ*	Half size of the body in *z* direction
*halfFootX*	Half size of the foot in *x* direction
*halfFootY*	Half size of the foot in *y* direction
*halfFootZ*	Half size of the foot in *z* direction
*L*_*f*_	Femur length
*L*_*t*_	Tibia length
*k*_*x*_	Spring stiffness in *x* direction
*k*_*y*_	Spring stiffness in *y* direction
*k*_*θ*_	Spring stiffness in *θ* direction
*k*_*step*_	Spring stiffness in the step phase
*λ*_*x*_	Damping coefficient in *x* direction
*λ*_*y*_	Damping coefficient in *y* direction
*λ*_*θ*_	Damping coefficient in *θ* direction
*standOffset*	Offset of the natural length of the virtual spring in the stand phase
*stepOffset*	Offset of the natural length of the virtual spring in the step phase
*stableTolerance*	Velocity threshold of the transition from the double support to the single support

In the standing phase, both feet are on the floor. We use the forward kinematics from the foot coordinate frame $\left\{B\right\}$ to the hip coordinate frame $\left\{C\right\}$ to calculate the pose of the hip.

$$X=\left[\begin{array}{c} x\\ y\\ \theta \end{array}\right]=\left[\begin{array}{c} L_{t}c_{a}+L_{f}c_{a+k}\\ L_{t}s_{a}+L_{f}s_{a+k}\\ \theta_{a}+\theta_{k}-\theta_{h} \end{array}\right]$$ (1)

Where (*x*, *y*) is the position of the hip, *θ* is the orientation of the hip, *θ*_*a*_ is the angle of the ankle, *θ*_*k*_ is the angle of the knee, *θ*_*h*_ is the angle of the hip, *L*_*f*_ is the femur length, and *L*_*t*_ is the tibia length. For convenience we represent the sine and cosine of *θ*_*a*_, *θ*_*a*_ + *θ*_*k*_ as *s*_*a*_, *c*_*a*_, *s*_*a*+*k*_, *c*_*a*+*k*_, respectively.

Then we can calculate the Jacobian by first-order partial derivatives of the pose with respect to each variable.

$$J=\left[\begin{array}{ccc} \frac{\partial x}{\partial \theta_{a}} & \frac{\partial x}{\partial \theta_{k}} & \frac{\partial x}{\partial \theta_{h}}\\ \frac{\partial y}{\partial \theta_{a}} & \frac{\partial y}{\partial \theta_{k}} & \frac{\partial y}{\partial \theta_{h}}\\ \frac{\partial \theta }{\partial \theta_{a}} & \frac{\partial \theta }{\partial \theta_{k}} & \frac{\partial \theta }{\partial \theta_{h}} \end{array}\right]=\left[\begin{array}{ccc} -L_{t}s_{a}-L_{f}s_{a+k} & -L_{f}s_{a+k} & 0\\ L_{t}c_{a}+L_{f}c_{a+k} & L_{f}c_{a+k} & 0\\ 1 & 1 & -1 \end{array}\right]$$ (2)

Finally, we use the transpose of the Jacobian and the virtual force to obtain the joint torques.

$$\tau =\left[\begin{array}{c} \tau_{a}\\ \tau_{k}\\ \tau_{h} \end{array}\right]=J^{T}F=\left[\begin{array}{ccc} -L_{t}s_{a}-L_{f}s_{a+k} & L_{t}c_{a}+L_{f}c_{a+k} & 1\\ -L_{f}s_{a+k} & L_{f}c_{a+k} & 1\\ 0 & 0 & -1 \end{array}\right]\left[\begin{array}{c} f_{x}\\ f_{y}\\ f_{\theta } \end{array}\right]$$ (3)

Where *τ*_*a*_, *τ*_*k*_, *τ*_*h*_ are the joint torques of the ankle, the knee, and the hip, and *f*_*x*_, *f*_*y*_, *f*_*θ*_ are the forces applied on the hip in the horizontal direction, the vertical direction and the rotation.

Due to the spring-damper components, we can obtain these forces by the following control laws.

$$\begin{array}{c} f_{x}=k_{x}\left(x_{d}-x\right)+\lambda_{x}\dot{x}\\ f_{y}=k_{y}\left(y_{d}-y\right)+\lambda_{y}\dot{y}\\ f_{\theta }=k_{\theta }\left(\theta_{d}-\theta \right)+\lambda_{\theta }\dot{\theta } \end{array}$$ (4)

Where *k*_*x*_, *k*_*y*_, *k*_*θ*_, *λ*_*x*_, *λ*_*y*_, *λ*_*θ*_ are the spring stiffness and the damping coefficient in *x*, *y*, and *θ* directions, *x*_*d*_, *y*_*d*_, *θ*_*d*_ are the desired position and the desired angle of the hip, and *x*, *y*, *θ*, $\dot{x}$, $\dot{y}$, $\dot{\theta }$ are the current position, angle, velocity, and angular velocity of the hip. In our work, we set the desired position *x*_*d*_, *y*_*d*_ as the original location, and set the desired angle *θ*_*d*_ as zero.

In the step phase, one leg needs to swing to take a step forward, so the joint torques of two legs are different. For the one whose foot is on the floor, the joint torques of the ankle, the knee, and the hip are the same as the standing phase, while for the swing leg, the joint torques of the ankle and the knee become zero, and the joint torque of the hip *τ*_*step*_ is applied by the torsional spring with *k*_*step*_ spring stiffness, as shown in figure 2, which is calculated by

$$\tau_{step}=-k_{step}\left(\theta_{d}-\theta \right)$$ (5)

The benefits of VMC are that it is compact, requires relatively small amounts of computation, and can be implemented in a distributed way. We could implement a high-level controller as a state machine that changes virtual component connections or parameters at the state transitions. Even though we use a discrete high-level controller, the overall motion can be smooth if the virtual components have a low-pass filter effect.

### 2.3 Walking State Machine

We choose a finite state machine (Syed, 2015) to conduct transitions between different states during the learning of walking. It allows the controller to use suitable virtual components for the current position and the walking cycle phase of the individual.

**FIGURE 3:**
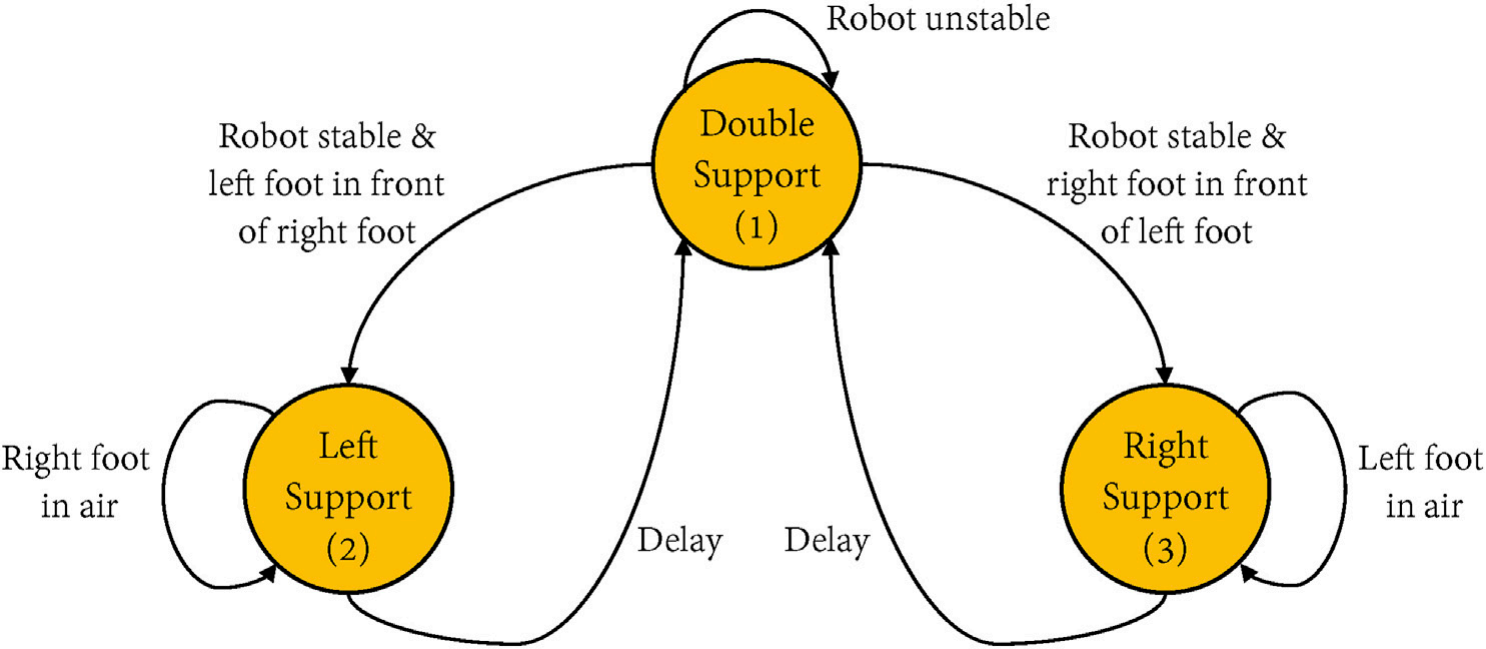
The finite state machine in the bipedal walking algorithm. There are three states during the walking cycle to allow transitions of different virtual components. The first state is the double support, which means both feet are contacting the floor, and the second state of left support means only the left foot contacting the floor. Similarly, the third state right support only has the right foot on the floor. The arrow indicates the conditions that need to be met for the transition. “Delay” means the delay time to allow for the swing leg to fall to the ground, so that the single support phase can transit to the double support phase. “Stable” means the creature has the possibility to walk forward, while in our work, we set a velocity threshold parameter *stableTolerance* to decide whether the angular velocity of the hip, the knee, and the ankle are smaller than the threshold. If they are all smaller than the velocity threshold, this situation is stable.

Figure 3 shows the finite state machine used in our bipedal walking algorithm, and there are three states: double support state, left support state and right support state. Table 2 lists the trigger events and the virtual components used in each state. The first state is the double support state. When the individual is stable, the state machine will check which foot is in front of the other foot. If the left foot is in front, the state machine will move into the left support state. In this state, the virtual components of the granny walker and swinging the right leg are activated. Similarly, the state machine will move into the right support state if the right foot is in front of the left foot, and the virtual components of granny walkers and swinging the left leg are activated. Since the swing leg is activated, both feet contact the floor, and the state machine will return to the double support state. The virtual granny walker is a mechanism with the vertical spring and the vertical damper which helps the creature maintain a constant height and regulate its pitch angle, while the virtual dogtrack bunny is a mechanism with the horizontal spring and the horizontal damper, which helps apply a virtual force in the forward horizontal direction to acquire the desired velocity, as shown in figure 2.

**TABLE 2:** Transitions of Walking State Machine.

State	Trigger Event	Virtual Component ^a^
Double Support	Delay after left or right support	VC1 & VC2
Left Support	Move right foot forwards	VC1 & VC3
Right Support	Move left foot forwards	VC1 & VC4

^a^Granny walker (VC1), Dogtrack bunny (VC2), Swing the right leg (VC3), Swing the left leg (VC4).

### 2.4 Simbody Simulator

Simbody is a high-performance, open-source C++ library providing sophisticated treatment of articulated multibody systems with particular attention to the needs of biomedical simulations. It is useful for predictive dynamic simulations of diverse biological systems such as neuromuscular biomechanical models and coarse-grained biomolecular modelling. It is also well suited to related simulation domains such as robotics, avatar simulations, and controls, and provides real-time capabilities that make it useful for interactive scientific simulations and virtual worlds (Sherman et al., 2011).

The simulation was conducted in a DELL OptiPlex 7,060 series desktop with Ubuntu 18.04 system, i7-8,700 processor, 12 threads, and 32 GB internal memory. We will terminate the learning process of the bipedal walking if the individual falls, while the upper body falls to below one half of the total body height, and it overruns a time limit of 15 seconds. The foot collision and ground reaction forces are realized by the Simbody simulator using *Collision Detection Algorithm* and *MobilizedBody*_*Ground* SimTK toolkits.

### 2.5 Performance Metric

Our ultimate goal in this work is to evolve independent bipedal walking in 15 seconds, where the fitness function aims to maximize the forward walking speed (the forward walking distance traveled divided by the total leg length). Here, we refer to the walking Froude number (Vaughan and O’Malley, 2005) to define the fitness function, where it is used to study trends in animal locomotion, and takes the total leg length into considerations.

$$Fitness=\frac{forward\;walking\;distance\;within\;15\;s}{total\;leg\;length}$$ (6)

It is important to note that in a simulation environment creatures can have drastically different sizes in length. It is equally easy to make a 1 m creature and a 1 km creature, and it would be very unfair if one step from the bigger creature was longer than 100 steps from the shorter one. In this case, the use of Froude number keeps an even field between creatures with different body structures.

Since we start the simulation at a supported tripod walking, there will be tripod walking individuals, bipedal walking individuals, and even individuals with alternating gaits. We use this behaviour as a metric to measure the performance of bipedal walkers. Also, we observe the growth rate of the fitness and the degree of body length decay as the other two performance metrics. We conduct the following three cases in different body length constraints in 4,000 generations. To verify the repeatability and the reliability of our results, we decide to do three replicates for each case.

#### 2.5.1 Free Body Length Scaffold

According to the current fitness, the genetic algorithm will choose the appropriate combination of body parameters and control parameters, benefiting from the algorithm. We let the body length evolve freely without additional restrictions. The body length constraint is set to as long as possible, which can provide as a scaffold to the biped creature, so that we set the upper bound of the body length as 1.8 m based on the leg length of the initial individual, while the lower bound is 0.05 m and equals to the diameter of the leg.

#### 2.5.2 Time-Constrained Body Length Scaffold

In order to analogize the gradual reduction of the external stability support for the body during the development of bipedal walking, we keep the lower bound as 0.05 m and restrict the body length by shortening the upper bound of the body length proportionally as the generations increase. The formula of the upper bound is as follow:

$$upper\;bound=1.8-0.4*i,\;i=\left\{\begin{array}{cc} 0\; & if\;G\in \left[1,500\right)\\ 1\; & if\;G\in \left[500,1000\right)\\ 2\; & if\;G\in \left[1000,1500\right)\\ 3\; & if\;G\in \left[1500,2000\right)\\ 4\; & if\;G\in \left[2000,4000\right] \end{array}\right.$$ (7)

Where *G* is the number of generations, the value of 1.8 is the body length of the initial individual, and the value of 0.4 is set as a decay coefficient.

#### 2.5.3 Performance-Based Body Length Scaffold

Considering the limitation of the number of generations, it is likely that the learning of bipedal walkers cannot be better searched. Therefore, we have balanced exploitation and exploration and designed a scaffold that limits the upper bound of the body length according to the current performance. The lower bound of the body length remains 0.05 m, which allows the algorithm to explore better at the beginning and focus on exploitation to maximize the performance. As for the calculation of the upper bound, we set a maximum operation between the performance-based scaffold and 0.05 (see 8). Therefore, the upper bound will never be less than the lower bound.

$$upper\;bound=max\left(1.8-0.4*\frac{AveBest3F}{InitF},0.05\right)$$ (8)

Where *InitF* is the fitness of the initial individual, *AveBest*3*F* is the average fitness of the current best three individuals, and the value of 1.8 and 0.4 are kept to be consistent with the time-constrained scaffold case.

## 3 Results

**FIGURE 4:**
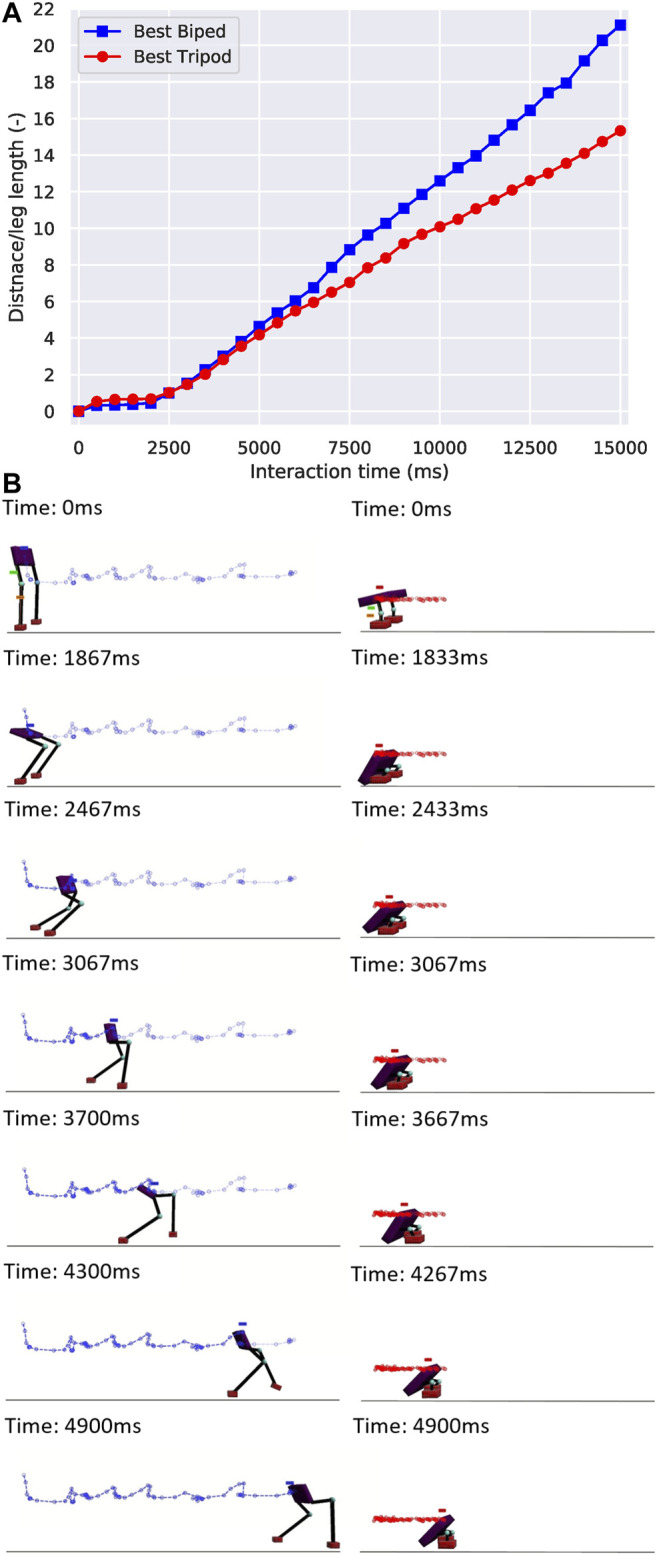
The fitness and snapshots of the best biped and best tripod creatures. **(A)** Fitness of the best biped and best tripod creatures over a 15 seconds interaction time, which is the creature interacting with the simulation environment per generation. The best biped has a femur length of 0.36 m and a tibia length of 0.81 m, while the values for the best tripod are 0.20 and 0.21 m. **(B)** The trajectory is generated by the motion analysis, with blue and red lines showing the path of the center of the body.

**FIGURE 5:**
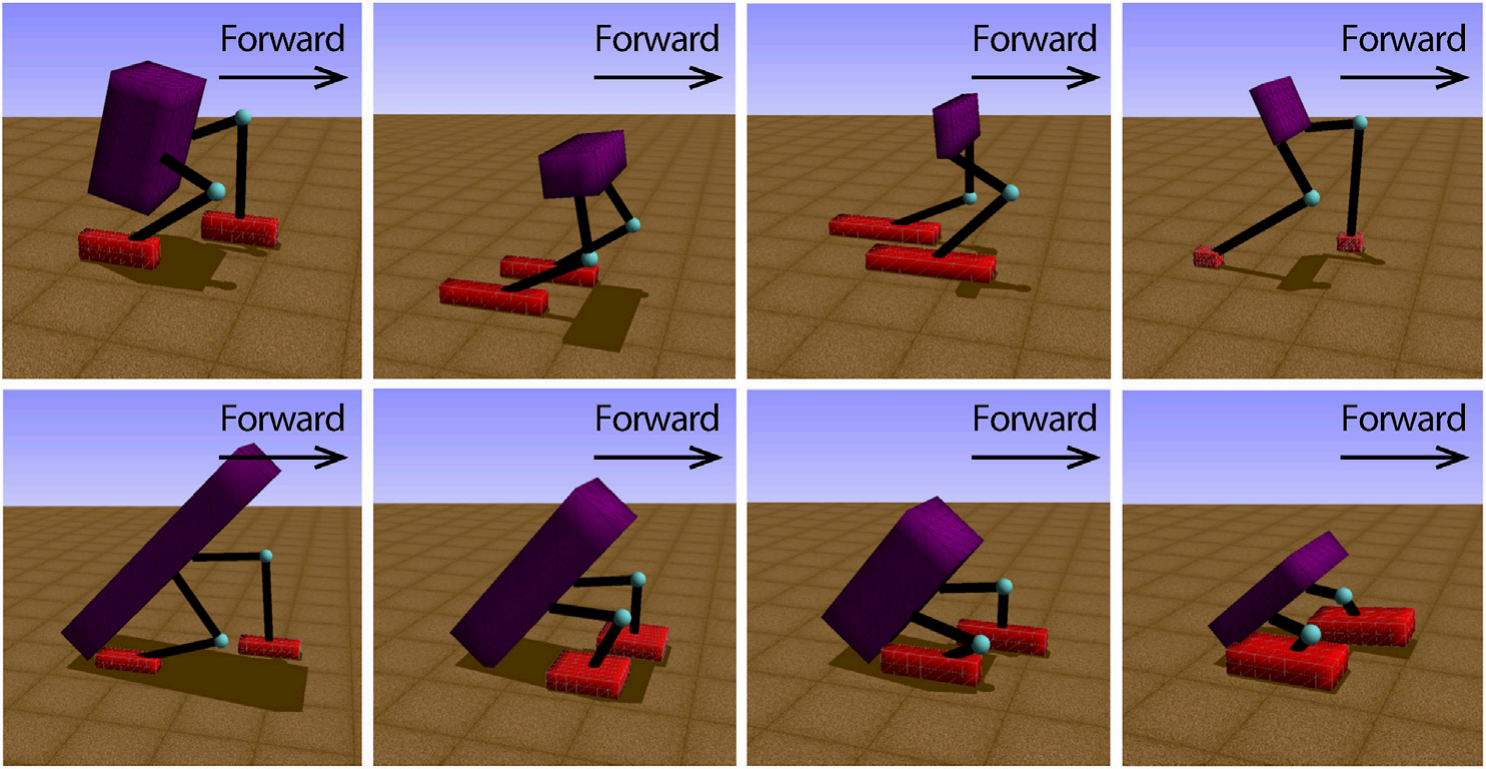
Individuals of bipeds and tripods with different leg lengths, body sizes and foot sizes evolved in the simulation. At the beginning of the generation, the length of the foot is very large, so that the foot has enough contact area with the ground, which benefits the individual to maintain stability. When the individual reaches the optimal bipedal gait **(the upper right figure)**, the short feet are more conducive to the swing of its legs and obtain faster forward speed. The upper figure of individuals are all bipedalism, and the lower figure of individuals perform supported tripod walking gaits.

After finishing all three cases, we started with a comparison between the fitness of the best biped and tripod from our simulations, as shown in figure 4*A*. Here, the best biped was found in the performance-based scaffold case and the best tripod was found in the time-constrained scaffold case. The interaction time is the creature interact with the simulation environment per generation, which is 15 seconds. Although these two creatures presented a very similar fitness initially, the biped gradually outperformed the tripod from the 4-s mark on-wards and reached a fitness value 37.5*%* larger than the tripod. We generated their body trajectories and plotted snapshots of these two creatures walking for 5 seconds (instead of the total 15 seconds), as shown in Figure 4B. From the figure, the stride length from the best biped was gradually increasing while the stride length of the best tripod was almost constant. In Figure 5, we demonstrated a variety of tripodal and biped creatures obtained during generations, and all their body lengths were gradually decreasing. Interestingly, we observed that most tripod creatures had long and broad feet, while the feet of biped creatures were continuously shrinking over generations.

**FIGURE 6:**
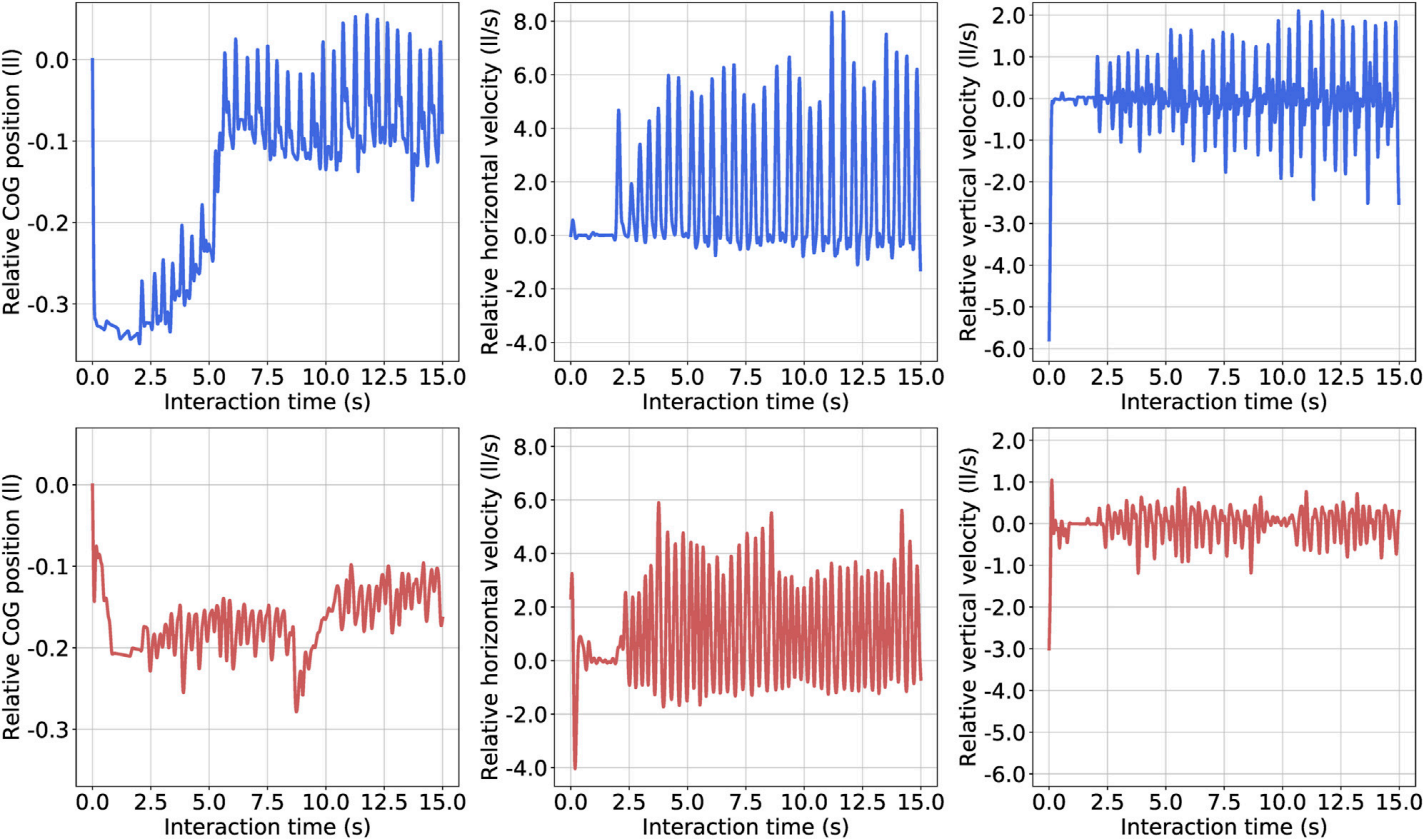
Gait analysis of best biped and best tripod. We pre-processed all data and divided it by the leg length to eliminate the inherent advantage of the biped creature with longer leg length than the tripod creature. The values have oscillations due to characteristics of the virtual model and the data captured at the center of the body. About the unit, *ll* represents the leg length, and *ll*/*s* represents the leg length per second. The upper three blue lines are the relative Center of Gravity (CoG) position, relative horizontal and vertical velocities of the best biped while the lower red lines are for the best tripod. Here, the relative CoG position is calculated based on the original CoG of the biped and tripod from the beginning of the simulation. For instance, 0 relative CoG position means the position of center of gravity at the beginning of the simulation.

**FIGURE 7:**
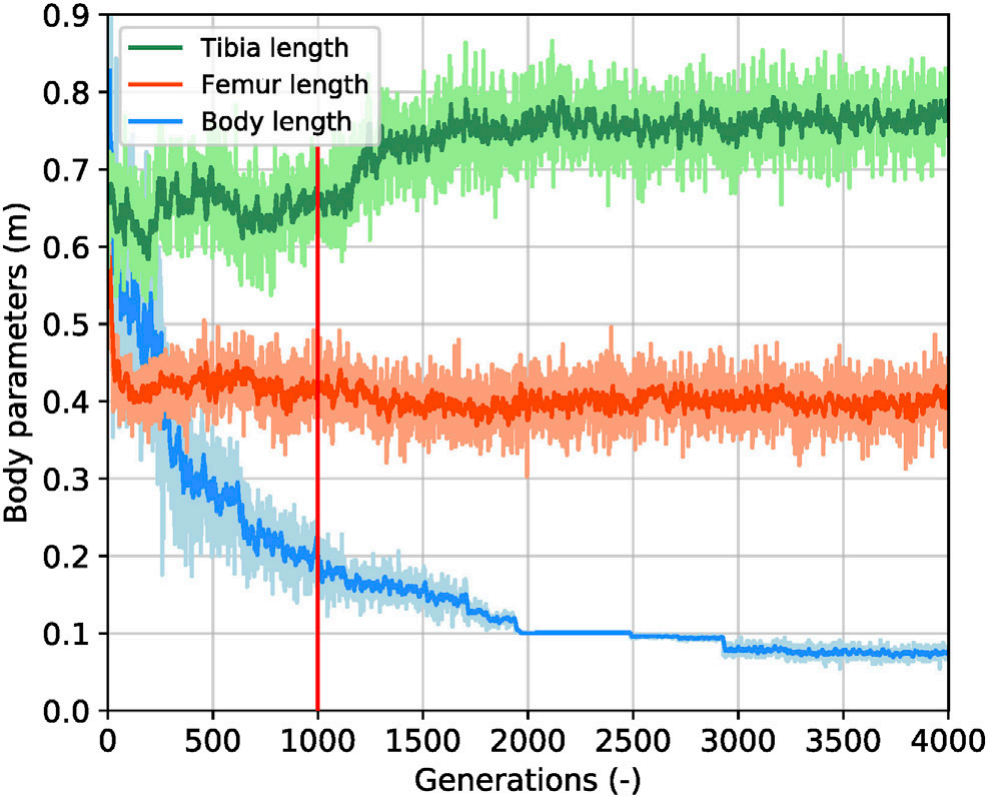
Parameters for the body length and the leg length over generations of the fast bipedal winner in the performance-based scaffolded case. The green curve and the orange curve are the tibia length and the femur length parameters, respectively. Their ranges are from 0.05 to 0.9 m, and they are set to mutate freely. The light-blue curve is the body length parameter which is forced to decrease based on the current performance observed, and the original range is from 0.05 to 1.8 m. Here, we squeeze the display range to 0.9 and use a smoothness function to draw the data clearly, which is conducive to investigate the changes of body parameters. The red line labels the place where the tibia length starts gradually increasing with the support of the body length.

We obtained the gait information from the relative Center of Gravity (CoG) position, the relative horizontal velocity, and the relative vertical velocity for both creatures, shown in Figure 6. Here, we defined the relative displacement unit as a leg length (*ll*), and the unit of the relative velocity as a leg length per second (*ll*/*s*) to fairly compare the locomotion from the best biped and the best tripod. Since we adopt a virtual model control method and capture the data at the center of the body, the value has some oscillations, such as the oscillation of the spring component of the virtual model. As for the CoG, the best biped first fell to -0.35 *ll* and returned to 0 *ll*, finally oscillating at the original position. The best tripod first fell to -0.2 *ll* and oscillated for the first 8 seconds. Then, it fell again to -0.3 *ll*, gradually rose back to -0.15 ll, and stabilized at that height. About the relative horizontal velocity, the best biped creature reached a maximum of eight *ll*/*s*, while the best tripod creature merely obtained six *ll*/*s*, so the relative horizontal velocity from bipeds was 30*%* higher than tripods. Regarding the relative vertical velocity, the best biped creature reached a maximum of two *ll*/*s* while the best tripod creature was half of it, at one *ll*/*s*. We plotted the variations that happened with body, femur and tibia length throughout the evolution of the biped creature, and we show it at Figure 7. The body length gradually decreases from 1.8 to 0.2 m over 1,000 generations, while the femur and tibia lengths had very few changes in this interim. After 1,000 generations, the body length continued decreasing, reaching 0.07 m, near the minimum value set in the simulation, while the tibia length increased 0.15 m and the femur length kept oscillating stably at 0.4 m. As shown in Figure 7, the body length rapidly decreases while the tibia and femur lengths are near-constant in the stability stage. It shows that the support for the body gradually shortens and loses contact with the floor, but the creature is still capable of walking forward. As the speed becomes faster, this method shows that the tripod walking converts to an unsupported biped gait.

**FIGURE 8:**
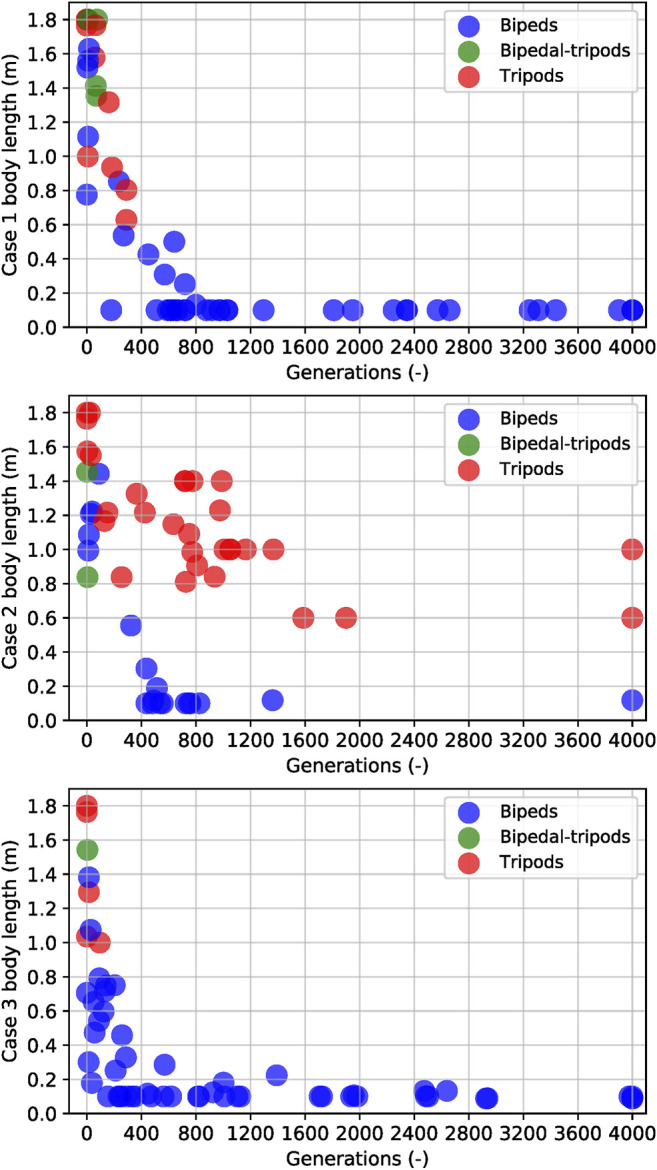
The scatter map of all three cases in three different trials with different body lengths at every bump of the current fitness. Case 1 is the body length with free constraint during generations, Case 2 is the constraint body length with decreasing value during generations, and Case 3 is the constraint body length based on the best fitness obtained during generations. Red stands for tripods, green for a hybrid bipedal-tripods, and blue for bipeds. Here, bipedal-tripod is the transition morphology between bipeds and tripods, while a creature walks supported with a long body occasionally. Since the simulations started from a tripod individual with the longest body length, the scatter map is red in the beginning. By growing with different body length constraint mechanisms, most individuals become bipeds (blue) at the end.

**FIGURE 9:**
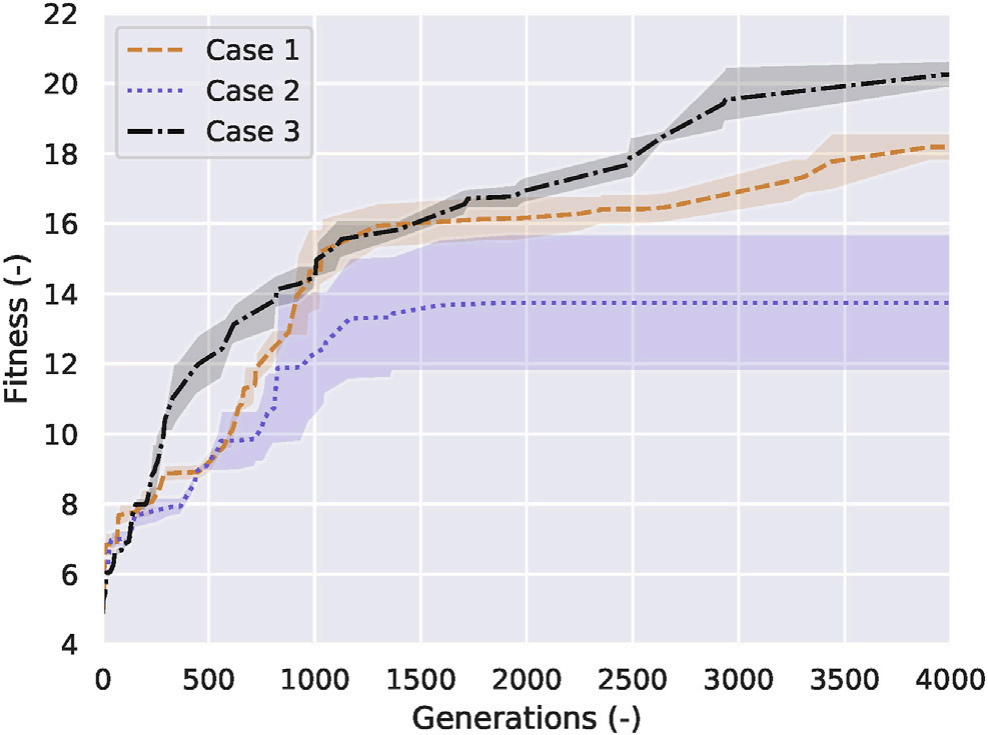
Results for all three cases within 4,000 generations. The median values of the brown dashed, purple dotted, and black dashdotted lines stand for free scaffolded learning case (Case 1), time-constrained scaffolded learning case (Case 2) and performance-based scaffolded learning case (Case 3), respectively. The shaded regions represent the standard deviation of each case. The variance of Case 2 and Case 3 are greater than Case 1 because we restrict the range of the body length of Case 2 and 3 based on the time and the current fitness, while we let the body length of Case 1 evolve freely.

The results described above were based on the best creatures, and those motivated us to create three cases (described at section 2.5) to understand the mechanisms leading to that difference. Initially, we wanted to identify the biped and tripod creatures in our simulation, and we plot Figure 8 to help us visualize the relationship between their gait and fitness. Over the course of 4,000 generations for each run, these creatures present a strong tendency to evolve from tripod to biped while also drastically decreasing their body length. We run each case three times, and observe that tripod creatures rely on a bigger body to support their gait, never reaching the minimum length possible. After averaging the results from those three trials for each case, we plot Figure 9, where we show the mean and standard deviation from each case. We observed that Case 1 (free scaffold) and Case 3 (performance-based scaffold) presented the bipedal results, while two-thirds of the runs from Case 2 (time-constrained scaffold) produced tripods as their best solutions.

In a comparison between Case 1 and Case 3 we can notice, from Figure 9, that the variance of the performance-based scaffold (Case 3) is greater than the free scaffold (Case 1) between 400 and 900 generations, but the learning curve in Case 3 is the steepest, and ultimately reaches the highest fitness. As for the difference of the variance, it is because we restrict the range of the body length of Case 3 based on the current fitness, while we let the body length of Case 1 evolve freely. Considering all the 4,000 generations, Case 3 is superior to Case 1 in 83*%* of the generations, even comparing the lower and higher bounds of the variances from both cases. Case 2, on the other hand, struggles to evolve and only reaches 66*%* of the fitness from Case 1.

## 4 Discussion

### 4.1 Body Length Supports Leg Growth

During the learning process of the best creature observed, the tripodal gait phase started with a long body and a short leg length, as shown in Figure 7. The definition of long and short for the body length and the leg length was relative to the optimal body parameters. Upon analyzing our results, we found that the creature experienced two stages before achieving the optimal parametric combination of body and leg lengths. From 0 generations to 1,000 generations, we observed a stability stage, with the body length rapidly decreasing from 1.8 to 0.2 m while the tibia and femur lengths were near-constant. The second stage, marked by a gradual speed increase, is defined by an increase in the femur length while the body length nearly reaches the minimum value set in the simulation.

In the first stage, the long body/short leg and its tripodal gait guaranteed the system to be stable to form a simple tripod control. Naturally, with an ever decreasing support, the short legs transition to a bipedal gait with a robust controller, and this triggers an increase in leg length to reach higher fitness values with an upright posture. This gait analysis allowed us to hypothesize on the internal mechanism of a scaffolded learning approach and strongly agreed with the work from Lungarella et al. (2003), where it is stated that roboticists could develop better systems by exploiting insights gained from studies on ontogenetic development. In this work, we state that A. stable tripodal gaits scaffold bipedal gaits and B. stable walking scaffolds speed increases, as seen in the transition from the first stage to the second, and our results are in strong agreement with a gait study with infants (Thelen et al., 1991). In this study toddlers who are still incapable of walking are supported on a treadmill and are capable of performing well-coordinated alternate stepping movements, in a very strong resemblance to an upright bipedal locomotion.

### 4.2 Performance-Based Scaffolds Bootstrap Learning

We proposed three cases of the scaffolded learning method in this paper. From the results shown in Figures 8, 9, we can state that the time-constrained scaffold (Case 2) hinders bipedal evolution, forcing the evolutionary process into a local optimum. On the other hand, a performance-based scaffold (Case 3) not only evolved bipedalism from tripods but also bootstraps its self-growth. Here, we set the free scaffold (Case 1) as the baseline of independent bipedal walking learning. Although Case 1 is better than Case 2, the learning curve seen in Case 3 is the steepest. Case 3 takes the correct cues to transition from tripodal to bipedal, by shortening its body length gradually to allow the controllers to mature, in strong agreement to the results shown in Rosendo et al. (2017); Zhu et al. (2019), where the role of morphology in the control development is studied. Case 1 is free scaffolded learning, and the freedom provided prevents the system from assigning a higher priority on the contribution from the body parameter. Case 2 shows the negative effects of a poorly conceived support system, where the controller for the creature over-matures at a longer body length and stagnating at a tripod gait in most of the times.

We can take the human ontogenetic development for the performance of a cognitive task as a child as the example of scaffolded learning cases. One is that parents deliberately do not interfere with the learning of their children, as seen in free scaffolded learning (Case 1), another is that parents slowly reduce their assistance for this child based on their age, as seen in time-constrained scaffolded learning (Case 2), and the other is that as this child performs this task parents adjust their support based on their perceived performance, as seen in performance-based scaffolded learning (Case 3). Broadening to pedagogical applications, Al Mamun et al. (2020) provides a positive example of how to implement inquiry-based learning in an online environment, considering the lack of direct teacher or peer support. However, they mentioned that recent research rises more attention as challenges increase when adopting a free scaffold in the self-regulated learning environment without direct support from teachers. Therefore, only by choosing a suitable method can we effectively accelerate the learning process, which is in agreement with our work of physical robots (Vujovic et al., 2017), where we show the negative effects of an improperly enforced developmental process on a robot.

## 5 Conclusion

In this paper, we introduced a scaffolded learning method on a creature capable of adapting its body and controller, hence bootstrapping a bipedal controller from a stable tripodal gait. Our results show that scaffolded learning with the optimal parameters is more productive than leaving a system free to learn independently. It is only true when the appropriate incentives behind scaffolded learning exist, effectively shortening the learning process with a performance-based scaffold, while a time-constrained scaffold is worse than the free learning case. Although bipedal walking can be reached through robust control methods, the study that we present here does not focus on the walking itself but on the capacity to use what is already known to bootstrap the unknown. We introduce a scaffolded learning method that accelerates the learning process, which can be combined with any learning method to improve the learning rate. We believe that the findings of this study are meaningful for machine learning in general, as our methods are not bound to genetic algorithms or one experiment, and could be adapted to different learning methods and different systems.

We would like to emphasize that this is the first time that such scaffolded learning method is used artificially, although pedagogy and cognitive scientists have observed animals and babies using scaffolds to support their learning processes, such as bike riders using training wheels or babies learning to stand while supporting themselves with chairs and sofas. In addition, this paper is not about locomotion, genetic algorithm, virtual model control, nor finite state machine, but about scaffolded learning being used to speed up a learning process, which can be used in any process and with any kind of learning algorithm. We use bipedal locomotion and tripod locomotion as a proof of concept for scaffolded learning. It could have been manipulation, jumping, standing or any other behavior that can have its initial steps supported by something. We propose the use of a structure combined with the software part, leaving the readers free to use a scaffolding method of their choice. As the field of Robotics suffers from the curse of dimensionality and the Reality Gap, our proposed method should be used on robots for faster deployment of learning algorithms and a bottom-up construction of this knowledge base. The same concept explained herein could be transposed to a simulation-scaffolded reality, with the eventual removal of the training wheels to reproduce a reality-compatible behavior. As is the case with humans, robots should also be capable of using their previously acquired knowledge to aid their learning of complex tasks. After all, if Newton could see further, it was by standing on the shoulder of giants.

## Data Availability

The original contributions presented in the study are included in the article/Supplementary Material, further inquiries can be directed to the corresponding author.

## References

[B1] Back T. (1996). Evolutionary Algorithms in Theory and Practice: Evolution Strategies, Evolutionary Programming, Genetic Algorithms. Oxford University Press.

[B2] Bril B. Brenière Y. (1993). “Chapter 13 Posture and Independent Locomotion in Early Childhood: Learning to Walk or Learning Dynamic Postural Control?,” in Advances in Psychology (Elsevier), 97, 337–358. 10.1016/s0166-4115(08)60959-0

[B3] Chaiklin S. (2003). The Zone of Proximal Development in Vygotsky's Analysis of Learning and Instruction. Vygotsky’s Educ. Theor. Cult. context 1, 39–64. 10.1017/cbo9780511840975.004

[B4] Chernova S. Veloso M. (2004). “An Evolutionary Approach to Gait Learning for Four-Legged Robots,” in 2004 IEEE/RSJ International Conference on Intelligent Robots and Systems (IROS)(IEEE Cat. No. 04CH37566) (IEEE), 2562–2567.

[B5] Giardina F. Iida F. (2016). “Simulation of Forward Hopping Dynamics in Robots and Animals Using a Template with a Circular Foot and Impulsive Actuation,” in 2016 6th IEEE International Conference on Biomedical Robotics and Biomechatronics (BioRob) (IEEE), June 2016, Singapore. 10.1109/biorob.2016.7523450

[B6] Giardina F. Mahadevan L. (2021). Models of Benthic Bipedalism. J. R. Soc. Interf. 18, 20200701. 10.1098/rsif.2020.0701 PMC7879758 33435842

[B7] Grillner S. Wallen P. (1985). Central Pattern Generators for Locomotion, with Special Reference to Vertebrates. Annu. Rev. Neurosci. 8, 233–261. 10.1146/annurev.ne.08.030185.001313 2984978

[B8] Hase K. Yamazaki N. (1998). Computer Simulation of the Ontogeny of Bipedal Walking. Bril and Breniè re: The Anthropological Society of Nippon, 106, 327–347. 10.1537/ase.106.327

[B9] Howard D. Eiben A. E. Kennedy D. F. Mouret J.-B. Valencia P. Winkler D. (2019). Evolving Embodied Intelligence from Materials to Machines. Nat. Mach Intell. 1, 12–19. 10.1038/s42256-018-0009-9

[B10] Kaipa K. N. Bongard J. C. Meltzoff A. N. (2010). Self Discovery Enables Robot Social Cognition: Are You My Teacher? Neural Networks 23, 1113–1124. 10.1016/j.neunet.2010.07.009 20732790

[B11] Lungarella M. Metta G. Pfeifer R. Sandini G. (2003). Developmental Robotics: a Survey. Connect. Sci. 15, 151–190. 10.1080/09540090310001655110

[B12] Mamun M. A. A. Lawrie G. Wright T. (2020). Instructional Design of Scaffolded Online Learning Modules for Self-Directed and Inquiry-Based Learning Environments. Comput. Educ. 144, 103695. 10.1016/j.compedu.2019.103695

[B13] Okamoto T. (1985). Human Infant Pre-independent and Independent Walking Primate Morphophysiology, Locomotor Analyses and Human Bipedalism. Tokyo: University of Tokyo Press.

[B14] Owaki D. Ishiguro A. (2006). “Enhancing Stability of a Passive Dynamic Running Biped by Exploiting a Nonlinear spring,” in 2006 IEEE/RSJ International Conference on Intelligent Robots and Systems, October 2006, Beijing, China (IEEE), 4923–4928. 10.1109/iros.2006.282452

[B15] Owaki D. Osuka K. Ishiguro A. (2008). “On the Embodiment that Enables Passive Dynamic Bipedal Running,” in 2008 IEEE International Conference on Robotics and Automation, May 2008, Pasadena, United States. (IEEE), 341–346.

[B16] Owaki D. Osuka K. Ishiguro A. (2013). Stabilization Mechanism Underlying Passive Dynamic Running. Adv. Robotics 27, 1399–1407. 10.1080/01691864.2013.839087

[B17] Pratt J. Dilworth P. Pratt G. (1997). “Virtual Model Control of a Bipedal Walking Robot,” in Proceedings of International Conference on Robotics and Automation (IEEE), April 1997, Albuquerque, United States, 193–198.

[B18] Quarles J. Lampotang S. Fischler I. Fishwick P. Lok B. (2009). Scaffolded Learning with Mixed Reality. Comput. Graphics 33, 34–46. 10.1016/j.cag.2008.11.005

[B19] Reil T. Husbands P. (2002). Evolution of central Pattern Generators for Bipedal Walking in a Real-Time Physics Environment. IEEE Trans. Evol. Computat. 6, 159–168. 10.1109/4235.996015

[B20] Rosendo A. Von Atzigen M. Iida F. (2017). The Trade-Off between Morphology and Control in the Co-optimized Design of Robots. PloS one 12, e0186107. 10.1371/journal.pone.0186107 29023482 PMC5638323

[B21] Saar K. A. Rosendo A. Iida F. (2017). “Bayesian Optimization of Gaits on a Bipedal Slip Model,” in IEEE International Conference on Robotics and Biomimetics (ROBIO) (Macau, China: IEEE), December 2017, 1812–1817. 10.1109/robio.2017.8324681

[B22] Schmidt N. M. Hoffmann M. Nakajima K. Pfeifer R. (2013). Bootstrapping Perception Using Information Theory: Case Studies in a Quadruped Robot Running on Different Grounds. Advs. Complex Syst. 16, 1250078. 10.1142/s0219525912500786

[B23] Sherman M. A. Seth A. Delp S. L. (2011). Simbody: Multibody Dynamics for Biomedical Research. Proced. Iutam 2, 241–261. 10.1016/j.piutam.2011.04.023 PMC4390141 25866705

[B24] Sims K. (1994). “Evolving Virtual Creatures,” in Proceedings of the 21st annual conference on Computer graphics and interactive techniques, July 1994, Orlando, United States. 10.1145/192161.192167

[B25] Susa Y. (1981). Dynamic Analysis of Infant Locomotion. Biomechanism 6, 59–68.

[B26] Swan K. R. Ives R. Wilson L. A. B. Humphrey L. T. (2020). Ontogenetic Changes in Femoral Cross-Sectional Geometry during Childhood Locomotor Development. Am. J. Phys. Anthropol. 173, 80–95. 10.1002/ajpa.24080 32656773

[B27] Syed A. (2015). Evolutionary Robotics Applied to Legged Locomotion. Bachelor Thesis.

[B28] Thelen E. Ulrich B. D. Wolff P. H. (1991). Hidden Skills: A Dynamic Systems Analysis of Treadmill Stepping during the First Year. New York: Monographs of the society for research in child development. 1922136

[B29] Vaughan C. L. O’Malley M. J. (2005). Froude and the Contribution of Naval Architecture to Our Understanding of Bipedal Locomotion. Gait & Posture 21, 350–362. 10.1016/j.gaitpost.2004.01.011 15760752

[B30] Vujovic V. Rosendo A. Brodbeck L. Iida F. (2017). Evolutionary Developmental Robotics: Improving Morphology and Control of Physical Robots. Artif. Life 23, 169–185. 10.1162/artl_a_00228 28513207

[B31] Vukobratovic M. Borovac B. Surla D. Stokic D. (2012). Biped Locomotion: Dynamics, Stability, Control and Application, 7. Springer Science & Business Media.

[B32] Wahde M. Pettersson J. (2002). “A Brief Review of Bipedal Robotics Research,” in Proceedings of the 8th UK Mechatronics Forum International Conference, June 2002, Netherlands (Amsterdam: Mechatronics), 480–488.

[B33] Zhu J. Li S. Wang Z. Rosendo A. (2019). “Influences of Incremental Mechanical Damage on the Bayesian Optimization of a Quadruped Robot,” in 2019 IEEE International Conference on Robotics and Automation (ICRA) Workshop on Towards Real-world Development of Legged Robots (IEEE), June 2019, Montreal, Canada.

